# Structures of the rat complement regulator CrrY

**DOI:** 10.1107/S1744309111016551

**Published:** 2011-06-23

**Authors:** Pietro Roversi, Steven Johnson, Joseph J. E. Caesar, Florence McLean, Kirstin J. Leath, Stefanos A. Tsiftsoglou, B. Paul Morgan, Claire L. Harris, Robert B. Sim, Susan M. Lea

**Affiliations:** a Sir William Dunn School of Pathology, Oxford University, South Parks Road, Oxford OX4 3RE, England; b Department of Biochemistry, Oxford University, South Parks Road, Oxford OX1 3QU, England; c Department of Infection, Immunity and Biochemistry, Cardiff University, Cardiff CF14 4XN, Wales

**Keywords:** complement regulator, CrrY, rat, CCP

## Abstract

Complement receptor 1-related protein Y (CrrY) is an important cell-surface regulator of complement that is unique to rodent species. The structure of rat CrrY domains 1–4 has been determined in two distinct crystal forms and reveals a 70° bend between domains 3 and 4. Comparisons of this structure with those of other complement regulators suggests that rearrangement of this interface may occur on forming the regulatory complex with C3b.

## Abstract 2

The structure of rat CrrY_1–4_ determined in two distinct crystal forms shows a pronounced bend at the interface between domains 3 and 4.

## 1. Introduction   

Complement constitutes the most ancient arm of the immune system, providing a first line of defence against blood infection by pathogens, and links innate to cellular immunity (Ricklin *et al.*, 2010 ▶). Prevention of activation on self-surfaces is achieved by complement regulatory proteins (Liszewski *et al.*, 1996 ▶). The two main complement regulation mechanisms that protect self-tissue from unwanted complement activation are decay-acceleration activity (DAA), in which the regulator dissociates the complement-activating C3 and C4 convertases, and factor I cofactor activity (CA), in which the regulator assists the serine protease factor I in cleaving and degrading the same C3 and C4 convertase precursors, C4b and C3b (Walport, 2001*a* ▶,*b* ▶).

Inappropriate or unregulated activation of complement has been implicated in a wide range of human inflammatory conditions (Szebeni, 2004 ▶). In order to investigate these conditions *in vivo* in mouse and rat models, the murine complement system and its regulators have been studied and characterized. These studies revealed that in addition to homologues of the human complement regulators CD46 (which has CA) and CD55 (which has only DAA), rats and mice possess an exclusively murine complement regulator: complement receptor 1-related protein Y (CrrY; Wong & Fearon, 1985 ▶; Foley *et al.*, 1993 ▶). CrrY is a membrane-bound protein comprised of five complement control protein (CCP) domains [also known as short consensus repeat (SCR) domains or Sushi domains] in mice and six or seven CCP domains in rats (owing to alternative splicing), followed by a transmembrane and cytoplasmic region in both rats and mice. The protein possesses both DAA and CA (Kim *et al.*, 1995 ▶; Li *et al.*, 1993 ▶).

CrrY is expressed across a broad range of tissues both in rats and in mice, while rat and mouse homologues of the membrane-bound regulators CD46 and CD55 (which are ubiquitously expressed in humans) are more tissue-specific. In mice CD46 is only expressed in the testis, while in rats expression is restricted to the testis and the acrosome of developing and mature spermatozoa (Mizuno *et al.*, 2004 ▶). The CD55 homologue in rats is widely expressed, with a tissue distribution similar to that of human CD55 (Spiller *et al.*, 1999 ▶). In mice, one CD55 homologue encodes a protein with wide tissue expression, while a second is only expressed in germ cells. Both CA and DAA are required to maintain homeostasis of the complement system and given the expression patterns of the CD46 and CD55 homologues CrrY is central to this process (Wu *et al.*, 2008 ▶). The importance of CrrY in controlling complement activation is highlighted by the embryonic lethal phenotype of CrrY^−/−^ mice (Xu *et al.*, 2000 ▶).

The first four CCP domains of rat CrrY (CrrY_1–4_) have been shown to have full complement regulatory activity (Fraser *et al.*, 2002 ▶). In this manuscript, we describe two crystal structures of a construct consisting of these first four CCP domains of rat CrrY and compare it with its functional human homologues CD55 and CD46.

## 2. Experimental   

### 2.1. Cloning, expression and purification   

A construct spanning the first four CCP domains of rat CrrY (CrrY_1–4_) was cloned and expressed in *Escherichia coli* BL21 (DE3) cells and refolded to allow formation of the eight disulfide bonds, as reported by Fraser *et al.* (2002 ▶). The sample was further purified by size-exclusion chromatography on an S200 16/60 column (GE Healthcare) in Tris-buffered saline (TBS). This material yielded crystals belonging to space group *P*2_1_2_1_2 (PDB entry 2xrd).

Unfortunately, the initial cloned construct was lost and cloning was reperformed without significant differences in the sequences (the new construct has one fewer Gly residue at the N-terminus and one extra Ser residue at the C-terminus). The purification protocol was also optimized to improve the sample purity and the newly cloned and purified protein yielded crystals belonging to space group *P*2_1_2_1_2_1_ (PDB entry 2xrb). The novel construct and protein-purification protocol are described in the following.

A cDNA clone of the rat CrrY gene was obtained from the mammalian gene collection (IMAGE ID 5599318) and a PCR insert encoding domains 1–4 was produced using the following primers (Sigma–Aldrich): 5′-GCCATCTACTCATATGCAGTGCCCAGC-3′ and 5′-GCGCTCGAGCTAGGATTTCACCTTGAAGCAGC-3′. This insert was ligated between the *Nde*I and *Xho*I sites of a modified pET-15b vector (Novagen) that has the *Nco*I site replaced with an *Nde*I site. The sequence was verified by sequencing.

CrrY_1–4_ was expressed in *E. coli* strain B834 (DE3) using Luria–Bertani broth in the presence of 100 µg ml^−1^ ampicillin. Large cultures were inoculated using a small overnight culture and were incubated at 310 K with rapid shaking. Protein expression was induced with 1 m*M* IPTG when *A*_600_ reached 0.6 and the cells were harvested by centrifugation (20 min, 5000*g*, 277 K) after 4 h of induction.

Cells from 4 l growth were resuspended in 40 ml 50 m*M* Tris, 1 m*M* ethylenediaminetetraacetic acid (EDTA), 150 m*M* NaCl pH 8.0 and lysed using an Emulsiflex C5 High Pressure Homogenizer (Avestin). 20 µl Tween-20 was added to the lysate, which was then rocked at 277 K for 5 min prior to centrifugation (20 min, 39 000*g*, 277 K). The supernatant was discarded and the pellet was resuspended in 40 ml 50 m*M* Tris, 1 m*M* EDTA, 150 m*M* NaCl pH 8.0. Again, 20 µl Tween-20 was added and the sample was rocked at 277 K for 1 h prior to centrifugation (20 min, 39 000*g*, 277 K). Inclusion bodies were solubilized in 20 ml 8 *M* urea, 100 m*M* Tris, 1 m*M* EDTA, 25 m*M* dithiothreitol (DTT) pH 8.5 and rocked at 277 K for 1 h. The sample was acidified to pH 3.5 using HCl and insoluble material was removed *via* centrifugation (20 min, 48 000*g*, 277 K). The supernatant was dialysed using 10 000 MWCO Snakeskin dialysis tubing (Pierce) against 6 *M* urea, 1 m*M* EDTA pH 3.5 overnight and any precipitate was removed by centrifugation (20 min, 48 000*g*, 277 K).

The supernatant was refolded by dropwise addition to 2 l 20 m*M* ethanolamine, 1 m*M* EDTA, 1 m*M* cysteine, 2 m*M* cystine at pH 11.0 at 277 K with constant stirring. The protein was then left overnight at 277 K to refold.

The volume of the refolded protein solution was reduced to 50 ml using a 10 000 MWCO Vivaflow concentrator (Sartorius) and the sample was then dialysed using 10 000 MWCO Snakeskin dialysis tubing (Pierce) against 25 m*M* Tris, 10 m*M* NaCl pH 7.4 overnight at 277 K. The protein was then concentrated to 10 ml in a 10 000 MWCO Vivaspin concentrator (Sartorius) and purified using an S75 16/60 size-exclusion column (GE Healthcare) equilibrated in 25 m*M* Tris, 10 m*M* NaCl pH 7.4. A final purification using a Mono Q 5/5 ion-exchange column (GE Healthcare) was performed using a protocol that started with binding in 25 m*M* Tris, 10 m*M* NaCl pH 7.4 buffer followed by a gradient reaching 25 m*M* Tris, 0.3 *M* NaCl pH 7.4 over 45 column volumes. CrrY_1–4_ eluted at 9.99 mS cm^−1^.

### 2.2. Crystallization and data processing   

Crystals of rat CrrY_1–4_ grew by vapour diffusion in sitting drops mixed at room temperature using an Oryx Nano crystallization robot (Douglas Instruments, UK). *P*2_1_2_1_2 crystals grew in 200 nl drops from a mixture of the CrrY_1–4_ protein stock at 6.6 mg ml^−1^ with mother liquor (2 *M* sodium chloride, 0.1 *M* sodium acetate pH 4.6) in a 7:3 ratio. Initial poor-quality crystals were optimized by streak-seeding and were cryoprotected with 30% glycerol. The *P*2_1_2_1_2_1_ rat CrrY_1–4_ crystals grew from a 1:1 mixture of CrrY_1–4_ at 3.3 mg ml^−1^ with mother liquor [0.2 *M* ammonium sulfate, 0.1 *M* sodium acetate pH 4.6, 30%(*w*/*v*) poly(ethylene)glycol 2000 monomethyl ether (PEG 2000 MME)] in 400 nl drops and were cryoprotected using 15% ethylene glycol. Diffraction data were collected on beamlines I04 at the Diamond Light Source (Harwell, England) and ID14-4 at the ESRF (Grenoble, France) and were processed with *XDS* (Kabsch, 2010 ▶) and *SCALA* (Evans, 2006 ▶) from within the *xia*2 data-processing suite (Winter, 2010 ▶). Data-collection statistics are gathered in Table 1 ▶.

### 2.3. Structure determination and refinement   

**Figure 1:**
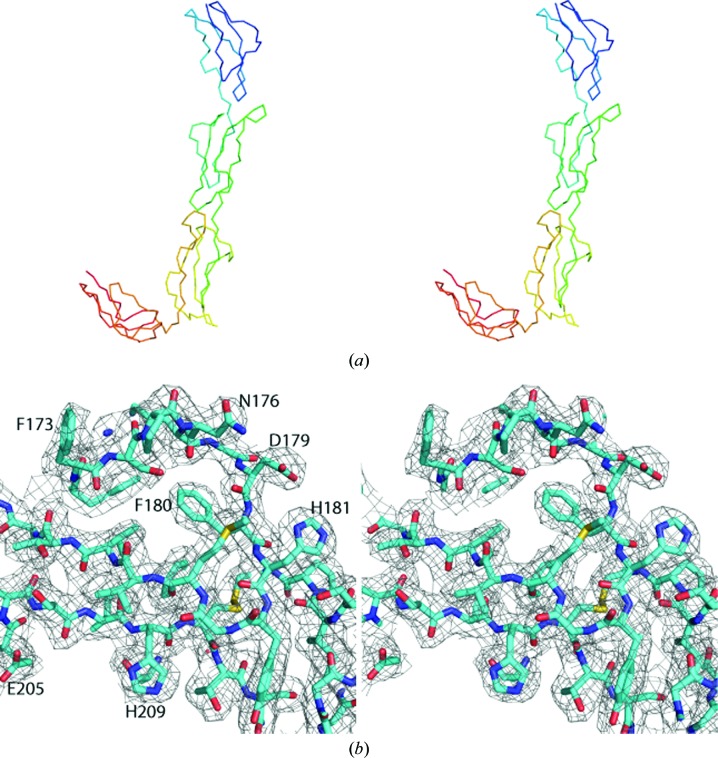
Stereographic images of the backbone of the CrrY_1–4_ structure and of a portion of the CrrY_1–4_ crystal electron density. (*a*) shows the backbone of CrrY_1–4_ coloured in rainbow colours: blue to red from the N-terminus to the C-terminus. (*b*) shows a representative sample of the electron density contoured at 1.0σ around Phe180. A few residues are labelled. This figure was made using *PyMOL* (DeLano, 2002 ▶).

The structure was initially solved in the lower resolution *P*2_1_2_1_2 form (PDB entry 2xrd) by sequential molecular replacement with the *CCP*4 (Winn *et al.*, 2011 ▶) program *Phaser* (McCoy *et al.*, 2007 ▶) using models of homologous individual domains [domain 2 of CD55 (PDB entry 1ok9; Lukacik *et al.*, 2004 ▶) and domain 15 of human CR1 (PDB entry 1gkn; Smith *et al.*, 2002 ▶)] trimmed using the program *CHAINSAW* (Stein, 2008 ▶). This model was then used to determine the structure of the higher resolution *P*2_1_2_1_2_1_ form (PDB entry 2xrb) by molecular replacement, again using *Phaser*. The structures were built and refined iteratively using *BUSTER*–*TNT* (Blanc *et al.*, 2004 ▶) and *Coot* (Emsley *et al.*, 2010 ▶), using secondary-structure target restraints in the lower resolution form (Table 2 ▶). Stereo images of a representative volume of the crystal electron density and of the entire protein main chain are shown in Fig. 1 ▶. The structures were deposited in the PDB with accession codes 2xrb and 2xrd.

## 3. Results and discussion   

**Figure 2:**
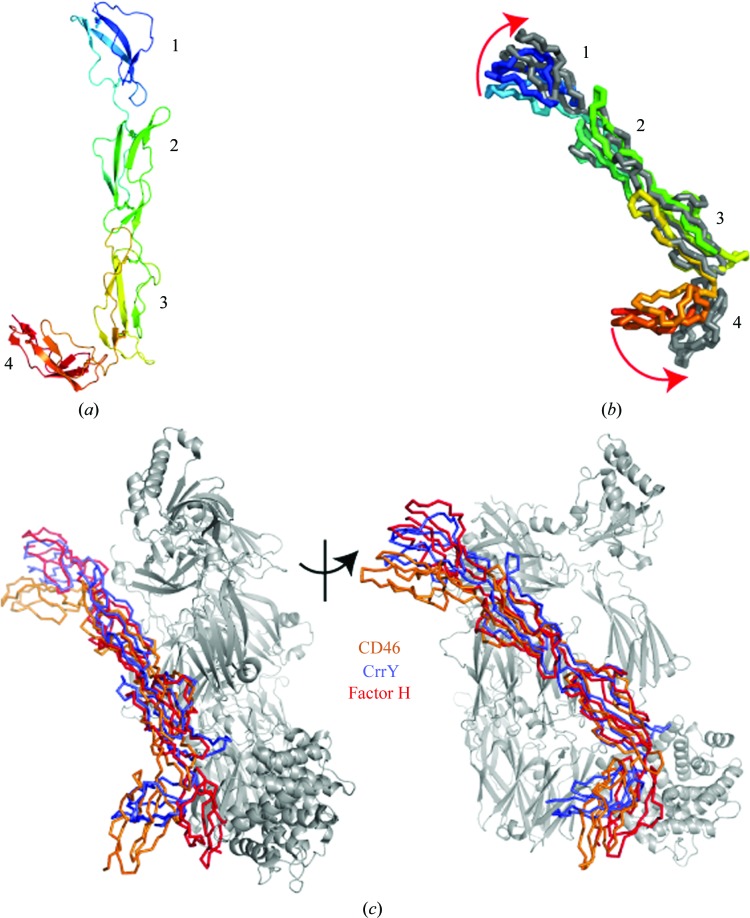
Factor I cofactors. (*a*) The crystal structure of the fI cofactor rat CrrY_1–4_ (PDB entry 2xrb) as a cartoon representation rainbow coloured from the N-terminus (red) to the C-­terminus (blue). (*b*) Overlay of the crystal structures of CrrY_1–4_ (PDB entries 2xrb and 2xrd; rainbow coloured) on the structure of fH_1–4_ from the C3b–fH_1–4_ binary complex (PDB entry 2wii; Wu *et al.*, 2009 ▶; grey). (*c*) Overlay of CrrY_1–4_ (blue, this work) and CD46_1–4_ (orange; PDB entry 3o8e; Persson *et al.*, 2010 ▶) on the crystal structure of the complex between fH_1–4_ and C3b (PDB entry 2wii). This figure was made using *PyMOL* (DeLano, 2002 ▶).

The two crystal structures of rat CrrY_1–4_ show a hockey-stick-shaped molecule with the elongated handle comprising CCP domains 1–3, with approximate dimensions 25 × 25 × 120 Å, and the blade made by CCP domain 4 (Fig. 1 ▶ *a*). The electron density of the crystal form diffracting to 2.5 Å resolution allowed unambiguous tracing of residues 37–290 (Fig. 1 ▶ *b*). The loop 52–54 is poorly ordered in the lower resolution structure. The domains are standard CCP domains and are organized in β-­sheets held together by two disulfide bridges (Fig. 2 ▶ *a*).

In both crystal forms rat CrrY_1–4_ retains the same overall conformation and secondary-structure elements, which can be taken to be representative of its solution structure. Small changes at the CCP1–CCP2 and CCP3–CCP4 interdomain junctions induced by the different crystal environments in the two crystal forms cause the two models to superpose with an overall r.m.s.d. on C^α^ atoms of 1.3 Å over 239 residues. The structural agreement within the individual CCP modules is closer, with r.m.s.d.s on C^α^ atoms of 0.53, 0.12, 0.29 and 0.60 Å for CCP1, CCP2, CCP3 and CCP4, respectively, over the ∼60 residues of each CCP domain between the two crystal forms.

**Figure 3:**
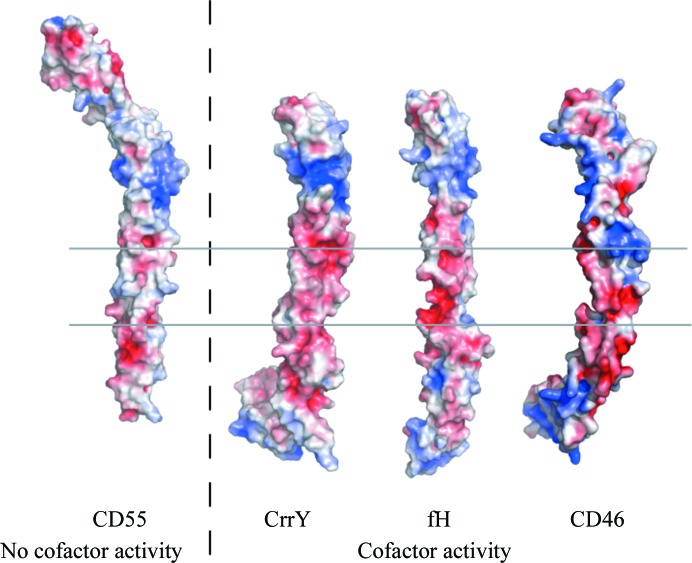
Electrostatic potential surfaces for CrrY_1–4_, fH_1–4_ and CD55_1–4_ computed with the *APBS* tool (Unni *et al.*, 2011 ▶) within the computer program *PyMOL* (DeLano, 2002 ▶). Contour levels: −3 *kT*/*e* (red) and +3 *kT*/*e* (blue). Shown are fH_1–4_ from the crystal structure of its complex with C3b (PDB entry 2wii), CrrY_1–4_ and CD55_1–4_ (from PDB entry 1ojv). CD55 is placed so that the functionally and structurally equivalent portion CCP2–CCP4 is aligned with CCP1–CCP3 in fH and CrrY (Harris *et al.*, 2007 ▶; Hocking *et al.*, 2008 ▶; Wu *et al.*, 2009 ▶). On the surface of the cofactors (CrrY_1–4_ and fH_1–4_) a strongly negatively charged patch is visible that is much weaker in CD55_1–4_ (which only has decay-accelerating activity).

The overall arrangement of CrrY_1–4_ is very similar to those observed in the crystal structures of other two factor I (fI) cofactors, fH_1–4_ (Wu *et al.*, 2009 ▶) and CD46_1–4_ (Persson *et al.*, 2010 ▶). The fH_1–4_ cofactor, as seen in the crystal structure of the C3b–fH_1–4_ complex (Wu *et al.*, 2009 ▶), overlays with CrrY_1–4_ particularly well in the first three domains, which are predicted to form the bulk of the contacts with fI (28% sequence identity, r.m.s.d. on C^α^ atoms of 1.6 Å over 180 residues). Fig. 2 ▶(*b*) shows both our crystal structures of CrrY_1–4_ overlaid on this structure of fH_1–4_. Similarly, the first three rat CrrY CCP domains, CrrY_1–3_, overlay with CD46_1–3_ with an r.m.s.d. on C^α^ atoms of 1.92 Å (34% sequence identity). Fig. 2 ▶(*c*) shows the overlay of CrrY_1–4_ with fH_1–4_ and CD46 in the context of the cofactor–C3b complex on the basis of alignment of domains 2 and 3. All three cofactors also share a patch of negatively charged residues on the surface that could contact factor I (Roversi *et al.*, 2011 ▶; see Fig. 3 ▶).

The rat CrrY_1–4_ CCP3–CCP4 interdomain interface, where the hockey-stick handle meets the blade, buries ∼300 Å^2^ and is centred on specific contacts between hydrophobic patches on CCP3 (residues 162–167) and CCP4 (residues 228–234 and 278–279), forming a kink in the structure. A kink was predicted at this interface by a solution-scattering study (Aslam *et al.*, 2003 ▶). The fH_1–4_ molecule in the crystal structure of domains 1–4 of fH (fH_1–4_) in complex with C3b also bends between CCP3 and CCP4 and this has been proposed to be crucial to function (Wu *et al.*, 2009 ▶). The structure of CD46_1–4_, recently determined in complex with a viral receptor also shows a pronounced bend at the same domain interface (Persson *et al.*, 2010 ▶). Thus, a kink at the interface between domains CCP3 and CCP4 in CrrY_1–4_, fH_1–4_ and CD46_1–4_ is likely to be a shared feature of all fI cofactors.

**Figure 4:**
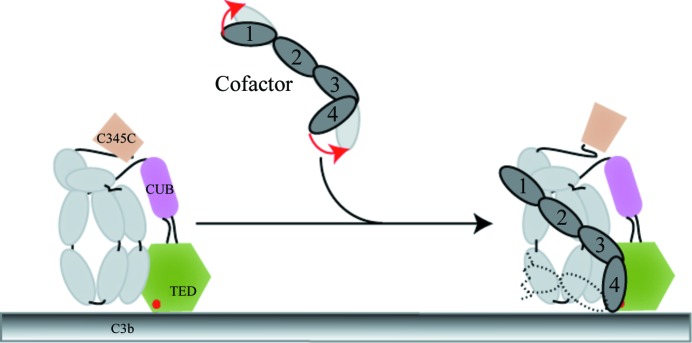
A cartoon illustrating the conformational rearrangement in the cofactor upon transition from the free unliganded state to the complex with C3b. The C3b representation is adapted from Wu *et al.* (2009 ▶). C345c domain, light bronze; CUB domain, pink; TED domain, light green (with the thioester as a red dot); LNK, α′NT and MG domains of C3b, light grey for Crr_Y1–4_, f_H1–4_ and CD55_1–4_. The functionally important cofactor CCP1–CCP4 domains are represented as ovals. Since several of the cofactor contain subsequent CCP domains, a few more have been sketched in dashed lines.

In the central portion of the regulator, the structure of fH_2–3_ overlays onto CrrY_2–3_ and CD46_2–3_ with r.m.s.d.s of 1.4 and 1.8 Å^2^, respectively, showing that CCP2 and CCP3 are not likely to change much upon going from free to bound regulator. The major differences between the free (CrrY_1–4_, CD46_1–4_) and C3b-bound (fH_1–4_) regulators localize at the CCP1–CCP2 and CCP3–CCP4 interfaces, which appear to be remodelled in forming the regulatory complex, bringing domains CCP1 and CCP4 closer to C3b. The change in orientation of CCP4 with respect to CCP2 and CCP3 is particularly striking when comparing the bound and unbound regulators, with CCP4 rotated more than 70° away from C3b in both the unbound structures (overlaid as described above) compared with the bound fH. In this context it is interesting to note that CD55, a regulator that does not possess cofactor activity, lacks a functional and structural equivalent of this fourth domain. We propose that the structural rearrangements at the CCP1–CCP2 and CCP3–CCP4 junctions upon binding to the C3b, as illustrated in Fig. 4 ▶, are key to formation of the cofactor–C3b regulatory complex.

## Supplementary Material

PDB reference: CrrY_1–4_, 2xrb

PDB reference: 2xrd

## Figures and Tables

**Table 1:** Data-collection and structure-solution statistics Values in parentheses are for the last shell.

Structure	2xrb	2xrd
Diffraction source	Diamond I04	ESRF ID14-4
Detector	ADSC CCD	
Temperature (K)	120	
Space group	*P*2_1_2_1_2_1_	*P*2_1_2_1_2
*Z*	4	4
Unit-cell parameters ()	*a* = 21.77, *b* = 105.34, *c* = 152.28	*a* = 205.48, *b* = 100.37, *c* = 21.95
Resolution ()	29.32.5 (2.62.5)	383.5 (3.73.5)
*R*_merge_	0.12 (0.53)	0.12 (0.45)
*I*/(*I*)	14.5 (3.8)	5.3 (2.5)
Completeness (%)	99.9 (100.0)	96.9 (94.0)
Multiplicity	6.8 (6.7)	3.0 (2.8)
Data-processing software	*XDS* and *SCALA*	
Phasing method	Molecular replacement	
Starting search model	1ojv domain 2 and 1gkn domain 15	
Alterations to search model	*CHAINSAW*	
Solution software	*Phaser*	

**Table 2:** Structure refinement and model validation

Structure	2xrb	2xrd
Refinement software	*BUSTER* *TNT* v.2.9.5	
Refinement on	*F*	
Resolution ()	29.262.50	383.5
No. of reflections	13030	6390
No. of reflections for *R*_free_	651 (125)	288 (75)
*R*_work_/*R*_free_	0.19/0.24	0.25/0.25
No. of atoms		
Protein	1966	1933
Ligand/ion	13 ethylene glycol (52 atoms), 3 SO_4_^2^ (15 atoms), 67 atoms in total	0
Water	125	0
*B* factors (^2^)		
Protein	31.9	68.2
Ligand/ion	49.8	
Water	35.1	
R.m.s. deviations		
Bond lengths ()	0.010	0.009
Bond angles ()	1.17	1.21
Ramachandran plot analysis		
Most favoured regions (%)	96	95
Disallowed regions (%)	0	0
